# Rapid-access oncodermatology is associated with reduced corticosteroid use and supports continuation of anticancer therapy after treatment-related cutaneous toxicity

**DOI:** 10.1016/j.jdin.2026.04.020

**Published:** 2026-05-19

**Authors:** Yuyang Liu, Samuel X. Tan, Nicholas M. Muller, Xiaohua Shen, Celine Lee, Yit Nah Lau, Victoria Atkinson, Rahul Ladwa, Emma-Anne Karlsen, Kiarash Khosrotehrani

**Affiliations:** a Department of Dermatology, Princess Alexandra Hospital, Brisbane, Australia; b University of Queensland, Frazer Institute, Brisbane, Australia; c Department of Medical Oncology, Princess Alexandra Hospital, Brisbane, Australia

**Keywords:** adverse events, checkpoint inhibitor, cIRAE, corticosteroid, cutaneous toxicity, immunotherapy, onco-dermatology, skin toxicity

*To the Editor:* Anticancer therapies are complicated by cutaneous toxicities, which affect up to half of patients on checkpoint immunotherapy.^1^ Presentations range from localized lichenoid or acneiform reactions to generalized bullous or erythrodermic eruptions requiring hospitalization. As per the Common Terminology Criteria for Adverse Events, cutaneous adverse events (CAEs) involving ≥30% of body surface area or limiting self-care activities are considered grade 3, and generally prompt therapy interruption or cessation.^2^ Even low-grade toxicities are frequently accompanied by xerosis and pruritus, which impair quality of life and contribute to patient-initiated withdrawal.^3^

CAEs are often managed with systemic corticosteroids (SCs). Although effective at short-term CAE resolution, immunosuppression may inhibit patients’ response to cancer, and SC exposure has been associated with lower response rates to checkpoint immunotherapy.^4^ There are no clear guidelines for multidisciplinary management of CAEs, and dermatology input is institution-specific and often underused.^5^ To address this, we present a pilot 18-month review of a rapid-access oncodermatology clinic, offering a steroid-sparing approach to promote therapy continuation following CAEs.

This retrospective study evaluated referrals to a public oncodermatology service in Brisbane, Australia (March 2024-August 2025). A total of 142 patients were included (median age: 66 years; 51% female), most frequently comprising melanoma (18%), breast cancer (16%), lung cancer (14%), and lymphoma (10%). Causative agents included anti-PD-1 monotherapy (28%), chemotherapy (16%), and anti-PD-1 combination therapy (11%). CAEs were graded as Common Terminology Criteria for Adverse Events grade 1 (33%), grade 2 (37%), or grade 3 (20%); 10% of referrals were unrelated to therapy, including skin cancer and cutaneous metastases (Fig 1).

**Fig 1:**
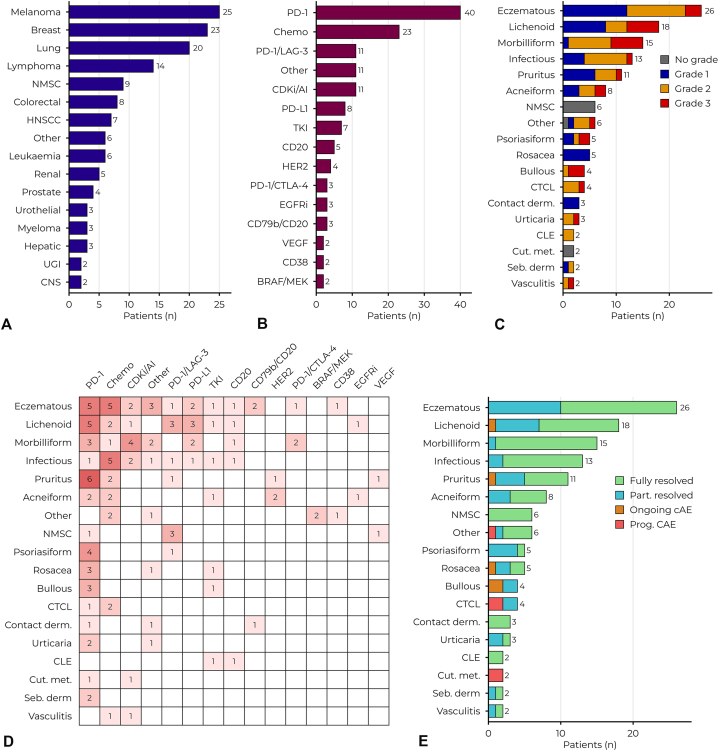
Pilot review of a rapid access oncodermatology service. **A,** Cancer type. ‘Other’ includes amyloidosis (1), anal squamous cell carcinoma (1), immune thrombocytopenia (1), and liposarcoma (1). **B,** Anticancer therapy at time of referral by mechanism of action. ‘Other’ includes therapies targeted towards CD3 (1), CD30 (1), HER3 (1), IDH (1), KMT2A (1), MDM2 (1), Nectin-4 (1), PD-1/TIGIT (1), and proteasome (1) inhibitors; as well as NSAA (1) and SERM (1). **C,** Cutaneous toxicities stratified by primary morphology and CTCAE grade. ‘Infectious’ includes bacterial (1), fungal (8), shingles (1), and scabies (3); ‘Other’ includes epidermoid cyst (1), erythema nodosum (1), miliaria (1), leukemia cutis (1), pityriasis rosea (1), and viral exanthem (1). **D,** Heatmap of anticancer therapy mechanism by cutaneous morphology. **E,** Outcome of cutaneous adverse events at 3-month follow-up. *CLE*, Cutaneous lupus erythematosus; *CNS*, central nervous system [cancer]; *Contact derm*, contact dermatitis; *CTCAE*, Common Terminology Criteria for Adverse Events; *CTCL*, cutaneous T-cell lymphoma; *Cut met*, cutaneous metastasis; *HNSCC*, head and neck squamous cell carcinoma; *NMSC*, non-melanoma skin cancer; *Seb derm*, seborrheic dermatitis; *UGI*, upper gastrointestinal [cancer].

Presentations included eczematous (18%), lichenoid (13%), morbilliform (11%), acneiform (9%), and psoriasiform reactions (4%) (Fig 1, *D*). Supportive management (72%), topical corticosteroids (57%), and antihistamines (29%) were frequently prescribed (Supplementary Table I, available via Mendeley at https://data.mendeley.com/datasets/n4xjv878wh/1). Oral antibiotics (15%) were applied for superimposed infections, acneiform rashes, and rosacea. Oral retinoids (5%) and narrowband UVB phototherapy (2%) were prescribed for immunotherapy-exacerbated psoriasis. Notably, the IL-4Rα inhibitor dupilumab was used in 3 grade 3 cases, comprising 1 eczematous eruption (complete resolution) and 2 bullous pemphigoid-type reactions (complete and partial resolution). Only 9% of patients required SCs, though this rose to 21% following grade 3 reactions.

Overall, 88% of CAE were completely (59%) or partially resolved (29%) within 3 months postreferral (Fig 1, *E*); 90% of grade 3 CAE were completely (36%) or partially resolved (54%). Dose reduction (4%), treatment interruption (12%), and cessation (8%) were rare. Cessation was required for only 14% of grade 3 reactions.

These findings support rapid-access oncodermatology as a reproducible service model, with 3 actionable items for multidisciplinary CAE management. Firstly, formal dermatology input was effective at relieving symptoms and quality of life in both low- and high-grade CAE. Secondly, dermatologist review in this cohort minimized avoidable or inappropriate SC use, which may otherwise reduce response rates to cancer therapy, aggravate conditions such as psoriasis, or exacerbate infections (eg scabies, dermatophytes) erroneously attributed to anticancer therapy. Thirdly, dermatologic biologics were effective at resolving severe CAEs in our review, and given their established safety profile may prove useful as steroid-sparing agents in the cancer setting.

## Conflicts of interest

None disclosed.
